# Probing neurodynamics of experienced emotions—a Hitchhiker’s guide to film fMRI

**DOI:** 10.1093/scan/nsad063

**Published:** 2023-11-01

**Authors:** Elenor Morgenroth, Laura Vilaclara, Michal Muszynski, Julian Gaviria, Patrik Vuilleumier, Dimitri Van De Ville

**Affiliations:** Neuro-X Institute, École Polytechnique Fédérale de Lausanne, Geneva 1202, Switzerland; Department of Radiology and Medical Informatics, University of Geneva, Geneva 1202, Switzerland; Swiss Center for Affective Sciences, University of Geneva, Geneva 1202, Switzerland; Neuro-X Institute, École Polytechnique Fédérale de Lausanne, Geneva 1202, Switzerland; Department of Radiology and Medical Informatics, University of Geneva, Geneva 1202, Switzerland; Department of Basic Neurosciences, University of Geneva, Geneva 1202, Switzerland; Swiss Center for Affective Sciences, University of Geneva, Geneva 1202, Switzerland; Department of Basic Neurosciences, University of Geneva, Geneva 1202, Switzerland; Department of Psychiatry, University of Geneva, Geneva 1202, Switzerland; Swiss Center for Affective Sciences, University of Geneva, Geneva 1202, Switzerland; Department of Basic Neurosciences, University of Geneva, Geneva 1202, Switzerland; CIBM Center for Biomedical Imaging, Geneva 1202, Switzerland; Neuro-X Institute, École Polytechnique Fédérale de Lausanne, Geneva 1202, Switzerland; Department of Radiology and Medical Informatics, University of Geneva, Geneva 1202, Switzerland; CIBM Center for Biomedical Imaging, Geneva 1202, Switzerland

**Keywords:** affective neuroscience, emotion, naturalistic stimuli, fMRI, fMRI analysis, film fMRI

## Abstract

Film functional magnetic resonance imaging (fMRI) has gained tremendous popularity in many areas of neuroscience. However, affective neuroscience remains somewhat behind in embracing this approach, even though films lend themselves to study how brain function gives rise to complex, dynamic and multivariate emotions. Here, we discuss the unique capabilities of film fMRI for emotion research, while providing a general guide of conducting such research. We first give a brief overview of emotion theories as these inform important design choices. Next, we discuss films as experimental paradigms for emotion elicitation and address the process of annotating them. We then situate film fMRI in the context of other fMRI approaches, and present an overview of results from extant studies so far with regard to advantages of film fMRI. We also give an overview of state-of-the-art analysis techniques including methods that probe neurodynamics. Finally, we convey limitations of using film fMRI to study emotion. In sum, this review offers a practitioners’ guide to the emerging field of film fMRI and underscores how it can advance affective neuroscience.

**Highlights**
- Film fMRI has grown in popularity for its unique capabilities to bridge the mismatch between emotion theory and affective neuroscience
- Film, as a paradigm, has shown numerous advantages over other methods of emotion induction and over traditional fMRI paradigms
- We provide an overview of relevant state-of-the art fMRI analysis techniques and their application to study emotion as complex, dynamic and multivariate phenomena
- In this review we advocate for a more systematic approach to study the neural underpinnings of emotion using film fMRI that is rooted in emotion theory

## Inception

With their 2004 study on inter-subject synchronization during film fMRI, Hasson and colleagues pioneered a new trend in neuroimaging to use films as a continuous stream of stimulation, instead of traditional experimental paradigms made of discrete conditions, to investigate brain functions with functional magnetic resonance imaging (fMRI) (Hasson *et al.*, 2004). Film fMRI offers a wide range of opportunities for the study of higher-order cognitive processes in (semi) naturalistic conditions, which cannot always be simulated in experimental studies (Nastase *et al.*, 2020; Jääskeläinen *et al.*, 2021).

Many areas of neuroscience have embraced the use of films, including vision (Nishimoto *et al.*, 2011), language (Hamilton and Huth, 2020) and clinical research (Eickhoff *et al.*, 2020). In all these areas, film fMRI helped to elucidate links between brain systems and cognitive functions. For example, in the area of language, film fMRI has been used to uncover a consistent atlas of semantic labels in the brain (Huth *et al.*, 2016). In vision, such an approach also contributed to demonstrating the distributed representation of object categories in the temporal cortex (Haxby *et al.*, 2020). Studies in affective neuroscience have also used film fMRI for over two decades; however, most of this research is limited to eliciting and measuring a few discrete emotions or dimensions at a time (Saarimäki, 2021), and it does not address temporal aspects underlying emotion experience (Horikawa *et al.*, 2020; Koide-Majima *et al.*, 2020). As transient mental and bodily episodes, emotions are intrinsically dynamic phenomena, both in terms of the eliciting events and context, and in terms of the unfolding of neural and physiological changes. More complex theoretical frameworks characterizing emotions as dynamic, multivariate and multi-dimensional are therefore valuable, but still rarely considered in this work. Yet, there is an opportunity to bridge the gap between complex models of emotion and feasible experimental paradigms, for which films are ideal, as they comprise rich dynamic stimulation and are designed to elicit a naturalistic and universal emotional response. Traditional task-based experimental paradigms, although very valuable, fall short in this respect as they, by definition, reduce the rich world of emotion experience to a small number of isolated, easily defined and comparable entities. By providing a much more naturalistic setting, film fMRI does not only address this mismatch between paradigm and research object, but also the discrepancy between highly controlled, simplified stimuli and brain processes that are designed to integrate complex real-world information (Sonkusare *et al.*, 2019).

Importantly, investigating emotion as a multivariate, dynamic phenomenon using fMRI is reliant on recent progress in fMRI acquisition, analysis techniques and computational power. (Bolton *et al.*, 2020a) provide a detailed overview of the ways brain dynamics can be probed in fMRI and how this has been applied to investigate particular behaviors or functions. Most notable are also the developments in fMRI analysis that stem from the emergence of resting-state fMRI research at the beginning of this century (Preti *et al.*, 2017; Hutchison *et al.*, 2013; Lurie *et al.*, 2020), as detailed by Finn (2021), without which film fMRI would not have the same potential as it does today.

Other outstanding reviews have already shown the advantages of using naturalistic stimuli in fMRI across a number of different domains (Sonkusare *et al.*, 2019; Vanderwal *et al.*, 2019; Eickhoff *et al.*, 2020; Hamilton and Huth, 2020; Jääskeläinen *et al.*, 2021). These reviews highlighted the vast progress owed to film fMRI in elucidating the neural mechanisms of psychopathology, language, cognition, emotion and development. Here, we will not provide another detailed description of neuroscientific findings on emotion using film fMRI; for this, we refer the reader to two recent and excellent reviews (Jääskeläinen *et al.*, 2021; Saarimäki, 2021). Instead, we aim to offer a practical guide on film fMRI methodology for the study of emotion, with the aim to enable and encourage its use for future research exploring emotion based on more comprehensive theoretical models. To this end, we provide an overview of relevant state-of-the art fMRI analysis techniques and their application to studying emotion as complex, dynamic and multivariate phenomena. We do not prescribe any one theoretical framework in particular, but stress their importance for scientific progress in the field and illustrate how they may guide empirical investigation with film fMRI, under the sole assumption that an appropriate framework should characterize emotions as multicomponential and dynamic in nature. Neither do we prescribe any specific approach or a unique set of experimental parameters, as doing so would be counterproductive to diversity and creativity in this research field. The purpose of this guide is rather to give an overview of the state of the art and tools available to investigate emotion experience using film fMRI, and thus equip researchers to fulfill their own goals and research questions. We hope that this approach will prove useful for the neuroscience community, even beyond emotion research, to fully leverage the potential of film in fMRI research.

## The three musketeers: emotion theories

**Fig. 1.**
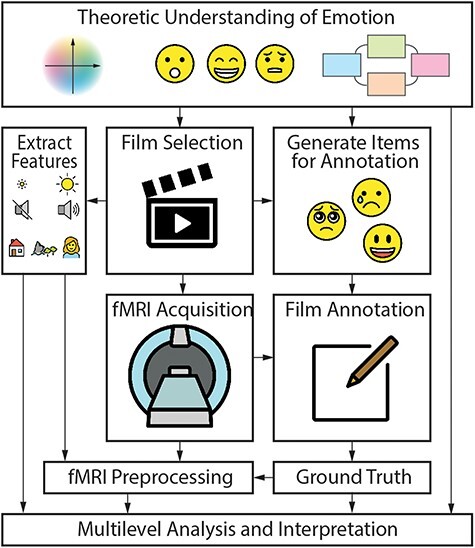
Research process for studying the neurodynamics of emotion with film fMRI. The beginning of the research process is to select on which theoretical framework (if any) the work will be based on. This will determine the choice of film material and features that will be annotated. When an independent sample is used, it is desirable to include some form of validation of annotations by the fMRI sample. From these annotations, a ground truth for each annotation item is calculated. fMRI acquisition can be conducted synchronously to the annotation process. After fMRI preprocessing, both behavioral and fMRI data may be analyzed at different levels of complexity. Emotion theory should inform the choice of analysis techniques and guide the interpretation of findings.

The scientific study of emotions demands a precise theoretical framework defining ‘emotion’ to assure clear research questions, select suitable methodological approaches and inform the interpretation of observations and further predictions (Levenstein *et al.*, 2023) (Figure 1). In traditional task-based research, the need for specific research questions is apparent early in designing a project, and one should not skip theoretical considerations when using film fMRI despite it being a task-free design. This section reviews three broad families of theories that have framed most fMRI work on the neurodynamics of emotions elicited by film.

The ‘discrete’ theories of emotions define affective phenomena as a set of genetically programmed and categorically distinct systems featured by prototypical facial (Ekman and Friesen, 1975) and bodily (Witkower and Tracy, 2019) expressions. Advanced analytical strategies on brain blood oxygenation level dependent (BOLD) data have revealed the neural underpinnings of a number of discrete emotions evoked by film clips (Peelen *et al.*, 2010; Hamann, 2012; Nummenmaa *et al.*, 2014; Jacob *et al.*, 2018; Ventura *et al.*, 2020), such as fear, disgust, joy and so on. These studies show patterns of distributed activation in partly overlapping cortical and subcortical areas to be involved in different types of emotion and have thus shifted our understanding away from emotion-specific engagement of local brain regions towards multivariate patterns (Kragel and LaBar, 2016a; Nummenmaa and Saarimäki, 2019), and complex network topologies with overlapping spatial configurations (Nummenmaa *et al.*, 2012; Alia-Klein *et al.*, 2020).

On the other side, dimensional theories describe emotions as psychophysiological states within a common functional space defined by independent variations in valence and arousal (Russell, 1980; Posner *et al.*, 2005). From this perspective, emotions emerge from psychological operations that are not specific to any affective experience but trigger specific degrees of valence and arousal. Thus, emotional categories are psychological constructs elaborated by domain-general systems processing different aspects of emotion experience, which are not unique to any emotional categories. Accordingly, fMRI studies have investigated the dynamic interactions of such domain-general structures in the brain and mapped distinct neural circuits encoding valence and arousal, while participants watched various films with emotional content (Kober *et al.*, 2008; Nummenmaa *et al.*, 2012; Wager *et al.*, 2015; Raz *et al.*, 2016; Chen *et al.*, 2018).

Lastly, ‘appraisal’ theories characterize emotions as the result of a continuous and recursive assessment of internally or externally driven information with regard to their relevance for wellbeing (Moors *et al.*, 2013). From this perspective, emotions are elicited according to how we evaluate a situation on multiple dimensions. The quality and intensity of our responses to this situation are accompanied by physiological responses and manifested in facial, vocal and gestural expressions. Initial appraisals, together with the physiological responses, are then integrated into a conscious affective experience—or ‘feeling’ (Scherer, 1984; Moors *et al.*, 2013). In this way, an emotion is not a single process but encompasses multiple components, engaged in sequence or in parallel, including appraisal processes, motivation processes, action and expression as well as physiology and bodily changes. Notably, this componential approach is a central framework for characterizing and modeling the intrapersonal operation of emotions in the field of affective computing (Gratch and Marsella, 2014). However, only few research questions have been investigated with film fMRI within this theoretical framework (Gaviria *et al.*, 2021; Mohammadi *et al.*, 2023).

Despite their conceptual discrepancies on defining emotion, these theoretical models also share some assumptions. First, there is consensus in neuroscience concerning a transmission of emotional phenomena over the course of evolution, as well as their essential role in adaptation (Anderson and Adolphs, 2014). Second, the dynamic nature of emotions, involving reactive and transient change in both mental and bodily states, is consistently highlighted in all theories (Kuppens, 2015). Third, mechanistic descriptions of how emotions are generated and organized in the brain are an important aspect considered to bring support to these models. Note also that different theories tend to emphasize partly distinct features of the same phenomena, with appraisal accounts focusing more on ‘input’ processes and differentiation, discrete accounts highlighting mainly ‘output’ processes and expression and dimensional accounts describing more internal feeling and physiology states. Accordingly, their different perspectives might be used to pinpoint different facets or constituents of emotion rather than considered as mutually contradictory.

In line with this, recent work using film fMRI suggests that an integration of concepts forged by different theories might provide broader descriptions of affective phenomena, for example by incorporating aspects from both discrete and dimensional theory in experimental designs and analysis (Kragel and LaBar, 2016b; Horikawa *et al.*, 2020; Koide-Majima *et al.*, 2020). Moreover, recent behavioral findings have shown, for instance, how emotions may be simultaneously conceptualized as discrete and dimensional (Cowen and Keltner, 2017; Lange and Zickfeld, 2021).

Finally, most fMRI studies attempting to map the neural basis of emotions have implemented a set of physiological and psychological features related to affective experiences. However, these features are not always integrated within theoretical frameworks that allow more defined topological descriptions of emotions in the brain. For example, studies may use one or more physiological variables (e.g. skin conductance) that are chosen as a generic label or a marker of an emotion variable (e.g. valence), but the relationships between these different measures are generally not explicitly conceptualized in precise functional or theoretical terms. Furthermore, the relationship between these variables is rarely considered when examining their effect on different brain activity metrics (e.g. BOLD amplitude or connectivity strength). These studies may thus fail to uncover how different psychological and physiological phenomena are mechanistically interdependent or integrated with each other. This makes it difficult to interpret some findings in the context of established theoretical frameworks that seek to explain emotion differentiation under general principles shared across various emotional states.

## Lost in translation: emotion elicitation and annotation

### The inside out of emotion during film watching

Many neuroscience studies have acknowledged the potential of films as a rich and efficient tool for emotional and cognitive stimulation (Nishimoto *et al.*, 2011; Hamilton and Huth, 2020). Films are designed to immerse their audience in a fictional reality with its stories and characters; watching them evokes a range of complex emotions of varying intensity that surpasses those evoked by more static stimuli (Martin, 1990; Uhrig *et al.*, 2016). Emotional events in films are expected to be well defined because they are generated by narratives with a precise and impactful architecture created by film directors. Empirical work annotating films with emotion labels has confirmed this assumption (Cowen and Keltner, 2017).

However, the nature of emotions evoked by films is specific to the spectator context; hence, the quality of these emotions might be distinct from emotions experienced in everyday life (Wiley, 2003). For instance, some appraisal theories include the concept of aesthetic emotions evoked by artistic productions, which are the result of an appraisal of the aesthetic appeal or virtue of the stimulus (Scherer, 2005; Silvia and Brown, 2007). They include emotions such as being moved, awe, admiration, rapture and solemnity. These emotions do not serve an obvious adaptive function in the same way as everyday emotions do. However, the quality of emotions evoked by films goes beyond aesthetic emotions when a viewer experiences a high level of absorption and identification with the portrayed events. This is why film experience can be intense and cover a wide range of emotions similar to those that are evoked by everyday life events.

A further distinction when researching emotions in the context of film is between first-person (experienced emotion) and third-person (portrayed emotion) experiences. Both have been extensively studied in the field (Labs *et al.*, 2015; Baveye *et al.*, 2015b; Uhrig *et al.*, 2016), but they reflect different research questions. For this review, we assume a primary interest in experienced emotions that are elicited in the viewer when watching an evocative scene or stimulus. It is important to instruct research participants to pay particular attention to their experienced emotion when annotating film material to ensure accurate reporting in this approach. Conversely, distinct and explicit instructions are required when participants need to appraise emotions experienced by others or any other emotional aspects of the films. Researchers may also choose specific kinds of films or introduce specific manipulations to determine how participants perceive the film stimulus, for example by using TV news with ‘real life’ footages or presenting the stimuli as ‘based on a true story’ that could enhance emotional responding. Instead, documentaries are usually intended to convey facts and less likely to evoke potent emotion responses, and hence they might actually be used as control conditions (Eryilmas *et al.*, 2011). In contrast, fictional film material appears ideal for emotion research as they are purposefully designed to immerse the audience in a shared, rich and diversified emotion experience. Nonetheless, specific instructions, such as to focus on the ‘playing’ or ‘faking’ of emotions of actors, may recruit emotion regulation strategies and reduce the affective impact (Vrtička *et al.*, 2011).

### The annotation game: implementing film annotation

One of the first steps of the research process using films is of course the selection and annotation of suitable material to evoke a range of emotions of interest. There are several criteria to consider for film stimulus selection, such as the film duration, its audiovisual content and the type and intensity of elicited emotions. One might also consider using lesser-known films and specifically recruiting subjects who have not seen the chosen films before, to avoid effects of repeat presentation. Selected film stimuli should be rich in emotional events that facilitate the viewer’s immersion in the narrative of the film. These events are typically intended to be non-ambiguous, but ambiguous events might also be considered when the resolution of conflicting emotions is envisioned or special film genres might be examined in light of individual habits or preferences (e.g. horror films). In addition, the richness of emotion elicitation could be exploited to interact with different personality traits and thus allow better understanding of inter-subject variability.

In practice, most emotion studies use either short clips each targeting one specific emotion or full-length films that are continuously annotated over time for the arousal-valence-dominance degree or in terms of discrete categories, allowing researchers to assess the emotional states evoked by different moments in the film (e.g. Baveye *et al.*, 2015a; Li *et al.*, 2015; Muszynski *et al.*, 2021). The advantage of full films over short excerpts that are emotion-specific is that the full context of a given emotional scene is presented to the viewer. Given the dependency of emotion experience on memory and appraisal of both previously and currently seen events, the context building up to an emotional event is of crucial importance to study affective processes in a naturalistic setting. When annotating full films or longer excerpts, emotions can be annotated for discrete sub-segments (e.g. time windows of a few seconds) or continuously (e.g. second by second).

We advocate for detailed annotations to be acquired in accordance with the theoretical account of interest, so as to shed light on the granularity and underlying functional constituents of emotions. These annotations can be acquired in a number of ways, as outlined in detail in a previous review (Saarimäki, 2021). An important consideration is also whether an independent sample is chosen to annotate films or the fMRI sample itself. There is a trade-off between capturing idiosyncratic responses to film events (every fMRI participant annotates the film after or during the MRI acquisition) *vs* modeling a universal emotional reaction (by determining a ground truth based on multiple annotators independent from the fMRI sample). This decision may also limit which methods and techniques are appropriate to use at the fMRI analysis stage.

When aiming to represent a ground truth of emotion evoked by a film, it is of central importance that these data are sufficiently validated. The assessment of annotation quality measured by inter-rater agreement is a key in the annotation process. When using discrete emotional annotations over time, Cohen’s Kappa coefficient can be used, whereas for continuous annotation time series, the concordance correlation coefficient (CCC) or Pearson correlation coefficient are computed (Muszynski *et al.*, 2021). Furthermore, with an increased number of annotators, the quality of annotations will be higher, and some studies suggested that at least six annotators should be involved to obtain reliable emotional characterization of stimulus content (Busso *et al.*, 2008). This number might, however, differ on a case-by-case basis, as in general, more annotators will be needed to achieve plausible agreement for more complex and subjective annotations. In any case, data from multiple annotators should be combined to calculate a ground truth for each annotation item. This can vary from calculating a mean time course across annotators to more sophisticated computations including normalizing, smoothing and weighting of raw annotation data (Li *et al.*, 2015; Baveye *et al.*, 2015b; Tian *et al.*, 2017).

More recently, efforts have been made towards extracting the emotional content of films using deep learning. Deep learning has drawn attention from the affective computing community because of its high performance in computer vision, speech processing and natural language processing tasks, such as object detection (Krizhevsky *et al.*, 2012) and sentiment analysis (Glorot *et al.*, 2011). Unfortunately, as for now, automated ways of emotion feature extraction are still very limited and fail to grasp the complex subtleties of emotional responses to films. Given the fast-growing popularity of deep learning and related fields, it is however worth addressing the possibility of using these methods to replace or supplement annotations from human raters in the future. While reliable automated feature extraction for emotions is currently not yet possible, deep learning approaches have already been successfully applied to other features of films that may be relevant to emotion research, such as the computer vision tools AlexNet (Krizhevsky *et al.*, 2012) and Oxford VGGNet (Xu *et al.*, 2014; Simonyan and Zisserman, 2015) or natural language processing tools (Devlin *et al.*, 2019). Deep learning approaches can thus be useful to code non-emotional (control) features from image, sound or language modalities, but they also have pitfalls due to (i) a large number of possible features can be extracted, (ii) many of those features are difficult to interpret and (iii) some features might be correlated with affective content. The fully data-driven procedure of these techniques may not allow any insight into the underlying processes behind the results obtained and their possible relation to brain functions. In addition, more advanced modeling will be required to extract other complex contextual features related to appraisal dimensions (Houlihan *et al.*, 2023). Nevertheless, despite a blackbox approach, deep learning and adjacent methods have become very popular and are expected to increasingly facilitate film annotations.

## There will be BOLD: films in fMRI research

Traditional fMRI designs are generally split into two categories: rest and task. Task fMRI uses a mixture of simple, controlled and often abstract stimuli arranged to study specific processes in isolation. Hence, a typical experimental task design alternates between task and control periods made of successively presented stimuli. Furthermore, participants are usually instructed to respond to the stimulus. In contrast, resting state fMRI observes spontaneous brain activity over continuous periods of time without experimental constraints nor active responses, revealing idiosyncratic patterns of static and dynamic functional connectivity (FC) that may predict behavior (Liégeois *et al.*, 2019) and vary across people, clinical conditions or even affective states (Du *et al.*, 2018; Gaviria *et al.*, 2021). Both methods have advantages and limitations. Whereas task paradigms are limited to a set number of experimental manipulations lacking ecological validity, but aiming to test specific theoretically derived processes, it is generally elusive which cognitive processes are engaged by individual subjects during scanning at rest and this can differ broadly between sessions. Rest, therefore, may thus result in rather noisy and highly variable data with limited interpretability and test-retest reliability.

Film fMRI can be conceptualized as an intermediate between task and resting-state fMRI. Similar to task fMRI, films elicit time-locked patterns of brain activation that are consistent between subjects (Hasson *et al.*, 2004). These patterns relate to features of the film or abstract meaning assigned to the film beyond sensory inputs, for example captured by annotations (Meer van der *et al.*, 2020). These features can thus be used to understand the neural underpinnings of specific cognitive functions engaged when watching the film. Brain responses to films have been found to be more consistent when stimuli have strong social elements and follow a non-ambiguous narrative (Meer van der *et al.*, 2020), which is an important notion to consider when selecting films for experiments. Moreover, in addition to these reliable response patterns, film fMRI can be approached as a continuous task-free modality in which subject-specific features in brain activation that can be analyzed in terms of static or dynamic FC are retained and used to predict behavior in the same way as resting-state fMRI.

Importantly, film fMRI provides better predictions of cognition and emotion than resting-state fMRI, potentially due to the richer variety and variability of brain states elicited during film watching (Finn and Bandettini, 2021). Due to the more constrained nature of film fMRI, it has a markedly greater test-retest reliability over rest, without compromising subject identifiability (Meer van der *et al.*, 2020). Finally, film fMRI also surpasses rest on fMRI compliance. Even when using an abstract film paradigm, less motion artifacts are measured and participants self-report less sleep compared to rest. This shows the power of films to absorb participants’ attention and the potential benefit for developmental and clinical research (Vanderwal *et al.*, 2015, 2019).

There are other specific features in films that make them a suitable stimulus to study higher cognition and affect in fMRI. Films present the viewer with an integrated audiovisual experience that traditionally follows a coherent narrative. The brain is especially sensitive to naturalistic information that unfolds over time, and therefore film stimuli offer a highly efficient tool to dissect functional brain networks in neuroscience studies. Although the visual system in our brain is finely tuned to complex sensory properties of input images, it responds even stronger to natural images than to systematic noise mimicking their sensory specifications (Puckett *et al.*, 2020). Similarly, it was shown that dynamic stimuli of faces evoke an increased brain response compared to static faces (Zinchenko *et al.*, 2018; Skiba and Vuilleumier, 2020). These effects are not limited to perception: film scenes with social and dynamic content are better suited to predict individual differences than scenes with static elements (Finn and Bandettini, 2021). In addition, some higher-order cognitive processes, especially those involving memory, are reliant on information unfolding over longer time scales, hard to capture with traditional fMRI paradigms. For example, it has been shown with narratives that different story lines have more distinct neural signatures over time (Chang *et al.*, 2021), or one can imagine how the quality of an emotion such as sadness over a character’s misfortune depends on the time invested in the said character. Thus, unsurprisingly, probing activation in brain areas implicated in such processes requires the presentation of stimulation developing over seconds or even minutes (Hasson *et al.*, 2008; Lahnakoski *et al.*, 2017). Few studies in affective neuroscience have exploited this temporal dimension of films to characterize emotion generation or differentiation, as proposed in psychology models (Moors *et al.*, 2013), and only artificial sequences of successive static stimuli have generally been used to manipulate the temporal dynamics of affective response (Kim *et al.*, 2004).

These considerations underscore the potential of film fMRI to assess specific brain circuits subserving dynamic mental processes that unfold over shorter or longer time windows, including emotion. Past research has unveiled a cortical temporal hierarchy of regions that are sensitive to information at different time scales from sensory to higher cognitive areas (Hasson *et al.*, 2008). A similar role of temporal trajectories within the space of relevant features or components is likely to underpin emotion experience, but it remains to be fully specified.

## Task fMRI unchained: analyzing fMRI data from film watching

The entire process of researching the neural underpinnings of emotion is informed by theoretical considerations as to the nature of emotion, and data analysis forms no exception to this. The key purpose of data analysis in this context is to integrate behavioral annotations and fMRI data to form a coherent understanding of emotion experience in the brain.

Depending on the theoretical framework underlying the research, there might be a considerable amount of behavioral data to be analyzed. This constitutes an essential step before fMRI analysis. In this review, we will focus on the analysis of continuous behavioral and non-neural physiological data only in conjunction with fMRI data; we refer the reader to other publications for purely behavioral analysis of complex emotion data (e.g. Borsboom *et al.*, 2021; Lange and Zickfeld, 2021).

Regarding fMRI preprocessing, standard pipelines as applied to task and rest fMRI can also be used for film fMRI (Nichols *et al.*, 2017). As is the standard in the field, we recommend to regress motion parameters, white matter and cerebral spinal fluid signals from preprocessed data. For other physiological signals, in particular heart rate and respiration, the situation is more complicated since these signals might be relevant to emotion elicitation, and thus these may be used as regressors of interest instead; e.g. increased heart rate may indicate arousal and affective state. On the other hand, by affecting blood flow and head motion, they might add to confounding noise effects. The decision on how to deal with these physiological parameters may also be guided by their importance in the theoretical framework being examined and the goal of a given experiment. The same applies to low-level features of the stimulus (e.g. luminance or loudness), which can be related to emotion elicitation (e.g. scenes that are intended to induce fear or suspense may include dim lighting and high contrast).

With respect to further analysis, film fMRI poses distinct challenges to data processing because most analysis techniques in fMRI have been optimized to either task or resting-state fMRI. Having situated film fMRI somewhere between task and rest, a combination of methods from both domains is favorable to explore the full complexity of film fMRI data. Nevertheless, there have been innovations in fMRI analysis specific to film fMRI, for example inter-subject phase synchronization (ISPS) and inter-subject correlation (ISC) (Hasson *et al.*, 2004; Simony *et al.*, 2016).

**Fig. 2.**
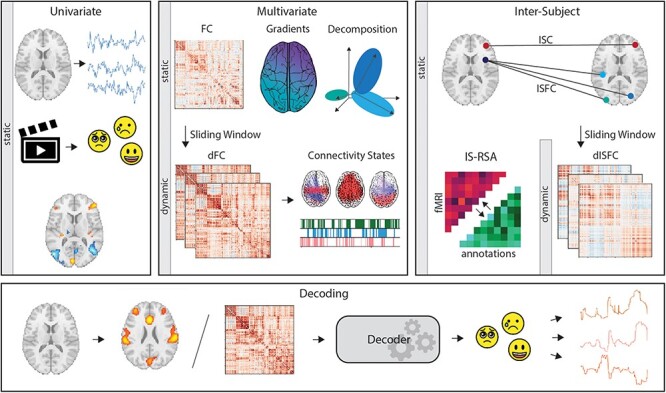
Analysis of film fMRI data to understand emotion experience. Analysis techniques range from static univariate to dynamic multivariate approaches which extract information on brain connectivity over time. Inter-Subject measures constitute a separate group of methods focused on shared activation between subjects. Finally, decoding can employ elements from different domains to reconstruct stimulus information (e.g. emotion experience) from fMRI data.

To specifically probe the neural mechanisms of experienced emotion from continuous film timeseries, a wealth of methods can be considered (Figure 2). We address these in order of complexity, as there is a progression from techniques focusing on spatial localization of functions through to multivariate techniques highlighting more distributed representations and finally dynamic approaches probing temporal aspects of emotion. We will also consider the capabilities of decoding approaches to classify and reconstruct emotion experience from fMRI data.

### Fifty shades of General Linear Model: mapping emotion experience

The most basic research question, once reliability and validity of the data has been ensured, is the localization of specific emotions or aspects of emotion in the brain. Mass univariate models are used to map the relationship between stimulus and magnitude of brain activation for each voxel independently. A very simple but powerful approach to functional mapping is the traditional General Linear Model (GLM) approach, which has been used in many previous studies focusing on small numbers of regressors (Qiao-Tasserit *et al.*, 2018; Sachs *et al.*, 2020). For this technique, annotations of the film stimuli are used as regressors to test which voxels are significantly related to their time course. As emotions can be influenced by audiovisual aspects of stimuli (Baveye *et al.*, 2015b), it is paramount to include relevant controls, for instance, semantic annotations and low-level properties of the film as nuisance regressors. GLM is a robust technique that may be used to narrow down global relationships between brain areas and specific emotion regressors. Moreover, GLM can also be used to test a computational model of behavior based on specific metrics, as it is commonly applied in reward and decision-making studies (O’doherty *et al.*, 2007). In that case, regressors are derived from the model instead of from directly observed behavior (e.g. see Leitão *et al.* (2020) for emotional responses during video-game playing). Nevertheless, this approach is less suitable for models with high collinearity or overparameterized models as it will hinder the estimability of contrasts, and thus has limited use for affective research. Furthermore, the classic GLM approach is not tuned to produce results that will generalize to other samples.

A solution to the methodological problems of the GLM approach is offered by voxelwise encoding models such as ridge regression. Ridge regression allows for a complex and high-dimensional space of emotions without the risk of overfitting due to the inclusion of a regularization parameter that consists of the sum-of-squares of the parameter weights. This parameter allows one to increase the number of regressors and explore complex relationships in a high-dimensional space of emotions. In much the same way as GLM, ridge regression provides information as to which voxels share significant variance with individual regressors. Researchers have successfully used this approach to show an isomorphism between the representation of emotion over the cortical surface and their relationships on a purely behavioral level (Lettieri *et al.*, 2019; Horikawa *et al.*, 2020; Koide-Majima *et al.*, 2020). Often in voxelwise modeling, methods derived from machine learning are used to create models of brain activity which can then be evaluated on an independent dataset (e.g. Huth *et al.* (2016)) or using cross-validation (e.g. Koide-Majima *et al.* (2020)).

Both GLM and Ridge Regression have been used in conjunction with multivariate methods to provide detailed maps of emotions onto the cortex as a semantic space or to understand differences in performance between models encoding emotion as categories *vs* dimensions (Kragel and LaBar, 2015; Horikawa *et al.*, 2020; Koide-Majima *et al.*, 2020). While this is a very simple approach, these kinds of comparisons between the neural representation of distinct aspects of emotion (e.g. emotions as discrete categories or dimensions) can give useful insights as to the merits of different theoretical frameworks. Thus, theories that predict discrete emotions to be an abstract description of a multicomponential experience may be investigated by comparing the neural signatures of different components *vs* that of a discrete emotion.

### Everything everywhere all at once: multivariate intra-subject measures

A major disadvantage of univariate methods is that they consider all voxels as independent and thus as unrelated entities. This is inconsistent with the nature of fMRI data in general but also more specifically with the fact that brain functions, such as emotion experience, arise in distributed functional networks composed of interdependent regions that are distributed across the cortex and subcortical structures (Fox *et al.*, 2005). Multivariate models allow for the prediction of multiple dependent variables, resulting in one outcome considering multivariate probability distribution. This means that unlike in mass univariate models, the activation of individual voxels is no longer considered as independent, thus giving the potential to unveil complex spatial patterns at play during film watching. Different approaches have been designed to capture distributed neural activity through inter-regional connectivity, which are described below and might be usefully applied to characterize changes in brain states during emotion episodes.

#### The usual suspects: static approaches.

##### The matrix: FC.

FC is defined as a measure of statistical interdependency between activity in two brain regions over time, most conventionally calculated by Pearson correlation. All functional connections in the brain, as a collective, form the functional connectome and can be represented in a brain graph as edge weights or as a matrix. FC is usually computed for regionally averaged time courses obtained during resting-state fMRI. In the context of film fMRI, FC can provide a broad idea of the relationships between brain regions during film watching. However, in its original form, FC is a static measure, and so it is most suitable to distinguish different fMRI runs, conditions or participant populations; consequently, its usability to encode emotion experience during film fMRI is limited due to the loss of temporal information in its calculation.

To achieve more meaningful interpretations, FC can be extended to account for context-dependent coupling, known as psychophysiological interaction (PPI) analysis (Friston *et al.*, 1997). In its original form, an interaction effect between the time course of a seed region and the paradigm (i.e. continuous emotion time course) is modeled to investigate its relationship to other brain regions. Thus, PPI is useful to understand FC that is specific to brain activation relating to a behavioral regressor. Furthermore, the generalized form of PPI (gPPI) allows for more than one regressor at a time (McLaren *et al.*, 2012). This means that with gPPI, film fMRI can be examined to determine which specific patterns of connectivity underly a particular emotion experience. However, this implies a priori choices concerning the relevant context or features of interest to be used for such regressors, based on the particular question or framework of the experimenter. Likewise, the choice of seed region is another a priori decision made by the experimenter and has important implications for subsequent analysis and interpretation. However, it is also possible to apply PPI to the entire functional connectome instead of a single seed region to extract task modulated connectivity (Di and Biswal, 2019).

##### Monty python and the holy gradient.

FC analysis provides an understanding of the brain’s intrinsic organization in networks. More recently, researchers have extracted macroscale smooth, ordered, gradual spatial changes in both structural connectivity and FC (Margulies *et al.*, 2016; Bijsterbosch *et al.*, 2021). This characterization of the connectome’s topography, also known as connectopic mapping or gradient analysis, is usually obtained by reducing the dimensionality of a similarity matrix of FC to extract spatial gradients (Haak *et al.*, 2018; Vos de Wael *et al.*, 2020). The obtained gradients describe leading whole brain organizational principles in terms of spatial dimensions. Gradient analysis has been met with great interest in naturalistic neuroscience, crucially as landmark findings in the field follow the topography of large-scale gradients (Huth *et al.*, 2016; Huntenburg *et al.*, 2018).

However, gradient analysis suffers from the same problem as conventional FC, namely the loss of temporal information. Nevertheless, it is useful to localize neural effects of emotion experience in the context of global cortical gradients defining functional brain architecture. In this respect, emotion experience has been shown to be encoded primarily in transmodal brain regions, which is consistent with the level of abstraction involved in emotion processing (Horikawa *et al.*, 2020). The application of gradients to film fMRI is still very recent and to date there is not much understanding of whether and how the organization of FC along gradients might be impacted by current emotion experience. Yet, there are clear indications that cortical organization differs during naturalistic stimulation compared to rest and thus that the present state of an individual is reflected in gradients (Samara *et al.*, 2023).

##### Million states baby: decomposition methods.

Dealing with fMRI data using all its spatial and temporal variables can be a limiting factor to many approaches. Therefore, the use of dimensionality-reduction techniques is often integrated into processing pipelines. Mathematically, the data are represented by a separable bilinear decomposition, i.e. a sum of components that each have a spatial (map) and temporal (time series) part.

The singular value decomposition (SVD) can be considered as one fundamental method to reach this aim as it directly acts upon the data matrix and produces variance-maximizing components that are orthogonal both in their spatial and temporal parts. These components can be linked to spatial or temporal principal component analysis (PCA), respectively. Depending on the viewpoint, the latent-space representation provides an efficient way to reduce dimensionality in space or time. The versatility of SVD/PCA is also often leveraged to reduce dimensionality of other types of data, such as emotion annotations. For instance, PCA can be performed as a second step after ridge regression to map many regressors onto a common multidimensional space characterized by PCA components. This has been applied to describe a continuous semantic space of emotion in the brain (Koide-Majima *et al.*, 2020).

Independent components analysis (ICA) is another popular decomposition applied to fMRI data that maximizes statistical independence between spatial components. In the context of emotion research, one can then use the regressed time series of each IC to explore their relationship with emotion experience. To date, there has only been one published attempt to apply ICA to film data and relate time courses of ICs to emotion experience, but the authors could not establish a reliable relationship between the two (Jääskeläinen *et al.*, 2008).

In contrast to SVD/PCA and ICA, partial least squares correlation (PLSC) analysis maximizes cross-covariance between two modalities and can thus directly account for emotion time courses or any other behavioral or physiological data, together with the fMRI data. The latent variables obtained in PLS thus represent the space that maximizes the cross-covariance between brain activity and emotion annotations. The resulting weights indicate how strongly each brain region or subject and emotion categories contribute to the multivariate brain-emotion correlation (Krishnan *et al.*, 2011; Lettieri *et al.*, 2019; Mohammadi *et al.*, 2023). While PLS is an elegant method to unveil cross-covariance between brain activation and emotion regressors, the results can be difficult to interpret.

#### Run Lola run: integrating emotion experience with brain dynamics

A key assumption about emotion that transcends theories is that emotions dynamically unfold over time. However, this assumption is often disregarded by the usage of analysis techniques that only probe spatial representation or connectivity averaged over time. Brain dynamics can be probed with a multitude of methods and investigating them may provide an important contribution towards unraveling the neural underpinnings of emotion (Bolton *et al.*, 2020a). Broadly, dynamic analysis techniques can be divided into sliding window and state-based.

FC, as measured by correlation between voxels or brain regions, can be extracted over a sliding window to obtain dynamic FC (dFC). Time courses of FC over the whole length of an acquisition are derived by evaluating the signal in consecutive time windows. There is some debate about optimal window lengths, though generally windows between 30 and 60 s are deemed to deliver meaningful fluctuations in dFC (Preti *et al.*, 2017). Changes in dFC are related to emotion experience; for example, studies found associations between intensity of tension, sadness, anger and fear with distinct dFC patterns (e.g. Raz *et al.*, 2012, 2016; Sun *et al.*, 2022).

Methods that use a sliding window to probe dynamics give a continuous measurement that can be directly linked to events in the stimulus. However, they face a trade-off between temporal resolution and reliability of their results. In contrast, clustering methods promise temporal resolutions up to that of the original sampling rate. While dFC can be clustered into distinct states assigned to each imaging frame, the temporal resolution achieved here only mimics to be frame-wise as the original measure is based on a sliding window. Other methods extract dynamic brain states based on a multitude of different metrics, chosen depending on research goals.

Importantly, irrespective of the method used, film fMRI data show greater variety in states compared to rest (Meer van der *et al.*, 2020), so it may be appropriate to choose more clusters than in standard resting-state fMRI when using methods where the number of states must be predefined. Traditionally, approaches describing discrete brain states produce summary measures, such as occupancy, duration and transitions for each state; however, another option is to reconstruct the respective brain patterns’ time courses to be used in subsequent regression analysis with emotion experience. Thus, it would be possible to determine how changes in emotion experience track with changes in brain states to disentangle the neural processes underlying specific emotions. In addition, it is also possible to extract information about overlapping states or the relative contribution of different brain states to each time point, which is especially meaningful to a multicomponential phenomenon such as emotion. Clustering techniques that allow for these data to be extracted seem promising to better understand the integration of different brain states over time to characterize the generation of emotion experience (Preti *et al.*, 2017). Notably, it is not yet an established practice in the field to consider time courses of states and evaluate overlapping states among simultaneous networks. Crucially, reconstructed time courses from state-based techniques typically only represent which state most closely matches the activation patterns at a given time point, and thus a finer detail that could be linked to specific events is lost. Therefore, to delineate the effects of closely related processes, this reduction to dominant clusters may not be the method of choice.

A series of clustering techniques use a subsample of the fMRI data to extract dynamic brain states. In co-activation patterns (CAPs) analysis for example, frames are selected when the whole brain or a chosen seed show strong amplitudes of activation. If there is a hypothesis regarding a particular brain region and its link to emotion, a seed-based analysis may be better than whole brain analysis as it reveals brain states specific to concomitant activation in this region (Gaviria *et al.*, 2021). Irrespective of the criteria for frame selection, CAPs are then derived by grouping the selected frames with a standard clustering technique (Bolton *et al.*, 2020c). A related approach is innovation driven co-activation patterns (iCAPs) analysis, which captures states related to transient changes in brain activation. Here, frames with transients in global signal are selected for clustering (Karahanoğlu *et al.*, 2013; Karahanoğlu and Van De Ville, 2015). Finally, a PPI-based frame selection can also be applied in what is coined PPI-CAPs (Freitas *et al.*, 2020). Here, frames can be selected when a BOLD signal increase is associated with emotion experience and clustered to derive states that capture the corresponding FC, explicitly related to a film-induced emotion. This technique allows for a more detailed differentiation between brain activations underlying similar emotions.

A richer clustering technique that has also gained traction in the field is variations of Hidden Markov Models (HMMs). In HMMs, the brain is considered to switch between discrete states over time, meaning that states do not overlap as each time point is assigned to one state only. The number of states extracted is decided upon a priori and therefore has a significant impact on the results. Importantly, HMMs are somewhat difficult to interpret and to relate to specific behavioral changes. Nevertheless, as with other state-based techniques, HMM-derived states can be linked to concurrent film annotations (Meer van der *et al.*, 2020). HMMs have also been used to characterize event transitions in naturalistic stimuli (Baldassano *et al.*, 2017). This technique has not yet been applied to emotion experience, but may be an interesting avenue to understand transitions in brain activity patterns as a function of affect.

### They came together: inter-subject measures

Inter-Subject FC (ISFC) is a method that relies on inter-region correlations between different subjects’ brains exposed to the same stimulus; this method offers much higher signal to noise ratio compared to conventional FC analyses, due to the removal of intrinsic neural dynamics and sources of noise that are uncorrelated across subjects (Simony *et al.*, 2016). Using this method, the signal induced by external stimulation (i.e. the film) is maximized, which is especially useful when working with regressors that may be inherently noisy, as those related to emotion (strongly correlated emotion components). Inter-subject measures are by definition assuming that similar appraisals will be engaged by similar events across different people and therefore biased to highlight functional processes and anatomical brain systems shared across individuals. While this is consistent with the assumption that emotional processes are subserved by similar brain networks shared across individuals (and goals of movie makers to elicit collective emotions in their audience), this approach obviously would not allow to fully address emotional phenomena in terms of constructivist theories that assign much weight to individual differences and circumstances. However, this does not necessarily prevent the fact that complementary approaches may be deployed to retrieve individual variations and map their possible substrates, on top of shared variance captured by ISFC.

In the same way as with FC, a sliding window can be applied to ISC and ISFC; thus, dynamic ISC or dynamic ISFC are obtained (dISC and dISFC). This allows for the identification of time-resolved changes in FC that are synchronized across participants and hence stimulus-driven. dISFC gives a dynamic measure of neural synchronization that can be related to changes in emotion experience over time either in its raw form (Nummenmaa *et al.*, 2012; Gruskin *et al.*, 2020) or following a dimensionality reduction such as k-means clustering (Bolton *et al.*, 2020b). Consequently, dISFC is useful to understand the dynamic patterns of brain activity driven by specific emotions irrespective of individual differences between subjects. Higher dISFC has been shown to be related to emotion intensity (Gruskin *et al.*, 2020), while different emotion dimensions such as valence or arousal drive synchronization in different brain areas (Nummenmaa *et al.*, 2012). The temporal sequence of inter-subject synchronization across the brain may also give insight into the chronology of processing that leads to the experience of an emotion.

As discussed above, sliding window approaches limit temporal resolution to the size of the chosen window, whereas ISPS provides a measure of inter-subject synchronization at the temporal resolution of the original fMRI sampling rate (Glerean *et al.*, 2012). Thus, the neural processes underlying changes in emotion can be linked to stimulus-driven changes in emotion experience in a more accurate and temporally informative manner. In the same way as with dISFC, yet with a higher temporal resolution, distinct brain areas have been shown to synchronize dependent on valence and arousal (Nummenmaa *et al.*, 2014).

Inter-subject measures can also be related to emotion experience by using Inter-Subject Representational Similarity Analysis (IS-RSA) (Finn *et al.*, 2020), which measures the degree of alignment between neuronal inter-subject similarities and behavioral inter-subject similarities. This can be used to localize shared activation and connectivity that are associated with shared emotion experience (Nummenmaa *et al.*, 2012, 2014; Jääskeläinen *et al.*, 2016). However, this technique relies on behavioral scores from each subject undergoing fMRI, which may not always be available.

Finally, more recent work has employed lag-ISFC, in which varying temporal lags are introduced between pairs of networks (Chang *et al.*, 2022). This technique probes the temporal sequence of activation in different functional networks. Lag-ISFC is a promising technique that has not been applied to study emotion experience, but may prove useful to understand the time scales of neural processes leading to an emotion. Because emotions are in essence defined as transient episodes in responses to particular events and their appraisals, triggering a cascade of adaptive neural processes, novel methods sensitive to dynamical transitions in brain activity might be particularly useful.

### Back to the emotion: decoding emotion experience from fMRI

The multitude of techniques described above have been used to describe various neural measures derived from BOLD as a function of emotion experience. Similarly, one can use these and other methods to decode emotions directly from brain activation. Decoding of diverse stimulus information from brain activation has shown promising capabilities in other fields within neuroscience. For example, deep convolutional neural networks have been used to successfully identify objects seen by the subjects (Horikawa and Kamitani, 2017) and films have been reconstructed using the so-called ‘motion-energy’ encoding model (Nishimoto *et al.*, 2011). Interestingly, the concept of FC has also been extended to characterize shared information between brain regions as locally revealed by pattern recognition, termed informational connectivity (Coutanche and Thompson-Schill, 2013). This approach has a broad applicability, including for memory processing (Bruett *et al.*, 2020), and it could help reveal how fine-grained patterns encode for information synchronously across the brain or how they relate to emotion differentiation during emotion unfolding. More information on the use of machine learning and deep learning for fMRI decoding, specifically on emotion, can be found in the work by Kragel *et al.* (2018).

In the field of affective neuroscience, promising results have been obtained when classifying a few basic emotions directly from brain activity using machine learning classifiers (Kragel and LaBar, 2015; Saarimäki *et al.*, 2016). Moreover, dozens of distinct emotions categories as well as affective dimensions were reliably predicted by both region and voxel-wise encoding models from film-fMRI data (Horikawa *et al.*, 2020). In isolation, these findings do not provide much insight into the neural mechanisms underlying emotion experience. However, based on a comparison between different theoretically informed decoding models, it was found that both categorical as well as dimensional aspects of emotion experience have predictive value and encode complementary information (Kragel and LaBar, 2015).

The field of deep learning requires a considerable amount of data that is difficult to come by in the relatively new field of film-fMRI. Additionally, neural networks do not allow for insight into the underlying processes that determine the results obtained, which poses a major limitation to their usefulness. Despite this black box aspect, deep learning and adjacent methods have become very popular and may make novel contributions to unveil the neural processes underlying emotion, especially when comparing models representing opposing theoretical frameworks.

## The good, the bad and the limitations

We have outlined the process of using film fMRI for emotion research and how advanced analysis techniques may be leveraged to understand the complex and dynamic neural processes underlying emotion in greater detail. Nevertheless, this kind of research is not easy and there are many pitfalls and sources of error that are discussed in the following section.

### Good emotion experience hunting

One of the main attractions of film fMRI is the potential to evoke an ecologically valid and naturalistic emotion experience. However, it is important to recognize that films are not designed to provide perfectly naturalistic stimulation. Films are primarily produced for entertainment. To tell a narrative in a compelling way, filmmakers can use frequent edits, leading to discontinuity in action, space and time, which the viewer must process to maintain a coherent representation of ongoing events (Magliano and Zacks, 2011). While commercial film editing is traditionally performed in a way to avoid major discontinuity, there is wisdom in choosing film material with minimal edits for research. Nevertheless, montage effects may contribute to the emotional impact of films, although they are not natural. Furthermore, unlike in a truly naturalistic setting, films do not provide a dynamic interaction between a stimulus and a subject. Stimulation is unidirectional and a passive perspective is imposed onto the viewer. Moreover, the MRI environment forces participants to remain motionless in supine position. This setting hinders the acquisition of reliable measures of active engagement with the stimulus. It is believed that films generally achieve high engagement, which is, for instance, evidenced by increased participant compliance during film fMRI (e.g. less motion and sleep). Participant engagement could additionally be measured through short self-report questionnaires after each fMRI run. More indirect measures of individual engagement might also be usefully explored, including eye fixation patterns, amounts of blinks or peripheral physiology markers. Nevertheless, for research questions that require strong first-person engagement and interactive behavior, film watching is not useful. Other approaches such as video games (Leitão *et al.*, 2020), hyperscanning for social interactions (Hamilton de, 2021) or virtual reality (Lenormand and Piolino, 2022; Somarathna *et al.*, 2022) may be superior in these cases. However, these approaches bear their own methodological hurdles.

Another key assumption of this research is that particular events in films evoke an emotion experience that is shared among viewers. This claim is supported by empirical findings and most films aim at and are successful in providing a strong shared emotion experience between audience members (Uhrig *et al.*, 2016). In fact, it has been shown that films are powerful in inducing both negative and positive emotional states in a consistent manner (Fernández-Aguilar *et al.*, 2019). What is not clear is to what granularity of emotion this remains the case. It is to be expected that some aspects of emotion experience are strongly correlated, but it is important to differentiate effects and determine how specific they are. Are films suitable to reliably distinguish aspects of emotion that are closely associated or frequently co-occurring? There does not seem to be a conclusive answer to this question at the time, but research employing multiple feature regressors show encouraging results alluding to fine grained differentiation between distinct aspects of emotions (Horikawa *et al.*, 2020; Koide-Majima *et al.*, 2020).

### White noise: limitations exacerbated in film fMRI

Predicting emotion from fMRI appears relatively harder compared to predicting other features from vision or cognition. One reason could be their dynamic nature and temporal unfolding (Kong *et al.*, 2019; Finn and Bandettini, 2021). Some of the analytical approaches using film fMRI as outlined in this review may prove useful to leverage dynamic information and overcome this limitation. Another reason is that there is still a lack of consensus on essential defining features of emotional phenomena (Scherer, 2005; Adolphs *et al.*, 2019).

There is a considerable lack of experimental control in film fMRI, compared to many standard lab studies. Firstly, there is no way to devise a true control condition. For memory or language research, it may be conceivable to use scrambled film clips to compare neural activity evoked by segments of different duration (Hasson *et al.*, 2015); yet, this seems incompatible for research on emotion experience as temporal context is such an integral determinant of emotion elicitation. It is further not possible to use repeat stimulus presentation as in traditional fMRI studies. For films, especially those with strong emotional content, it is expected that repeated presentation will evoke distinct brain responses so the usual benefit of repeat presentation does not apply. This limits conclusions that can be drawn from film fMRI data, as it impedes estimating the proportion of variance explained by the stimulus (Horikawa *et al.*, 2020). Another confounding factor to be on the watch out for is that specific low-level features of film may be related to specific emotions; for example, fear eliciting scenes are often darker or louder (Baveye *et al.*, 2015a,b; Saarimäki, 2021). Despite these limitations, film fMRI can act as its own internal control if sufficient data with events of varied emotion intensity are available for each subject. Furthermore, low-level features and semantic annotations should be considered.

It is generally agreed that subcortical structures are central to several neural processes pertaining to emotion, including not only the amygdala but also hypothalamus, brainstem or deep gray nuclei in the brain. However, activation in these structures is difficult to measure due to their small size and location close to diverse tissue types, especially larger blood vessels (Boubela *et al.*, 2015). Furthermore, their internal organization is complex and comprises intermingled neuronal populations that may overlap anatomically but contribute to distinct or even opposite affective processes functionally (Pignatelli and Beyeler, 2019; Li *et al.*, 2022). In contrast, network analyses of film fMRI often rely on surface-based descriptions of brain organization that tend to focus on cortical areas and neglect subcortical structures. Here, we advise caution and refer to advances in fMRI acquisition and analysis to carefully consider how to mitigate lower signal-to-noise ratio in subcortical regions. Faster acquisition sequences and the advent of ultra-high field fMRI can improve imaging subcortical regions (Kim and Bandettini, 2023). Furthermore, different methods of denoising the fMRI signal during preprocessing, including considerations of brain anatomy and physiological noise, have been shown effective (Maugeri *et al.*, 2018).

We also believe that more technical developments are needed to this avail. Subcortical areas are likely to make important contributions to emotion processes and should be carefully considered in future studies employing film fMRI with novel methodologies.

### A space odyssey: temporality

Most emotion theories imply predictions regarding the temporal sequence of how an emotion is generated following a stimulus. Often this includes very fast temporal relationships between emotion components (Lewis, 2005; Moors *et al.*, 2013). The temporal resolution of fMRI, constrained by the hemodynamic response, is likely too low to detect precise temporal relationships. This relates to a further constraint regarding temporality. Films with very fast transitions between events can lead to overlapping effects in the fMRI signal that are very hard to untangle and assign to specific events in the film. Hence, it is recommended to avoid choosing films that rapidly change context. Nevertheless, advances are being made to allow capturing changes in BOLD at much faster time scales than traditionally though possible; firstly by updating how the hemodynamic response is modeled, and secondly by utilizing multiband sequences in acquisition to achieve shorter repetition times (Polimeni and Lewis, 2021). These are promising developments for the field. However, one may consider using other acquisition methods such as magneto- or electroencephalography when pursuing specific research questions related to fine-grained temporal aspects of emotion. These techniques remain rather underexploited in emotion research, and methods to combine data from different modalities are still limited.

In addition to the mismatch of temporal resolution between emotion experience and fMRI measurements, there may be considerable differences in the time scales at which different emotions and emotion components unfold. For example, by definition, surprise occurs very suddenly, while love is expected to slowly build up and decrease over time. These differences in time scales may cause issues with dynamic techniques based on sliding windows, but also with framewise methods (see also above). It is important to be aware of the expected time scales of events of interest, and how temporal smoothing of any kind or transitions in the fMRI signal may be affected by this. These differences in time scales are mirrored in the temporal features of neural processes. Hasson *et al.* (2008) define temporal receptive window (TRW) as the time period during which a brain region is affected by a stimulus. Brain regions responsible for low-level processes such as perception are characterized by very short TRWs, whereas those involved with higher-level processes such as memory or cognition will have longer TRWs (Bartels and Zeki, 2005; Hasson *et al.*, 2015).

A final important aspect regarding the temporality of film fMRI is that the viewers’ mental representation and consequently their emotion experience are tied to the chronology of the film. Any event during a film happens in the context of what has occurred before. This process of a chronologically expanding mental model of narrative and characters needs to be synchronous between the fMRI subject and the annotators. Thus, whenever possible, annotations should be collected for entire films at a time, rather than for isolated scenes. Furthermore, interpretation of specific film events and their associated neural patterns must respect the temporal context of the event.

### Ordinary people: individual differences

Individual differences are recognized as an important factor determining emotion by different theories, particularly constructivist and appraisal theories (Lewis, 2005). Unsurprisingly, individual differences account for a considerable part of the variation in emotion experience and the corresponding brain response (Okon-Singer *et al.*, 2013). Often, brain regions and networks associated with higher-order cognitive and affective processing show highly idiosyncratic activation during film watching, revealing the importance of subject-specific variables to understand the functioning of these areas (Hasson *et al.*, 2004; Golland *et al.*, 2007). Particularly when working with heterogeneous participant groups, it is important to consider individual differences. Many of the presented analysis techniques can be modulated to allow for individual subject variation to be factored either in or out. However, individual differences are equally complex and multifaceted as is emotion. Consequently, integrating all levels of individual differences when researching emotion as a complex and dynamic phenomenon may be near impossible with the current technologies and computational capacities.

While individual differences are undoubtedly an important factor determining emotion, we feel that a first major step for affective neuroscience to improve understanding of the neuronal foundations of emotion experience should begin with what is shared between subjects, at least within socially and culturally homogenous groups. For this reason, we have not considered variation between subjects in this review. Please note, however, that some theoretical models and annotation parameters that can be applied to analyze film fMRI data (such as those associated with contextual appraisals) may already provide useful insights into specific emotion components or dimensions that are most likely to be influenced by individual factors such as personal values or moral norms (Sander *et al.*, 2003; Cunningham and Brosch, 2012).

Nonetheless, understanding the effect of individual differences remains an important direction for future research. This is particularly true in the context of affective disorders as having a clearer understanding of how emotion processes are disrupted in patients can open new pathways for treatment. Several of the methods reviewed above might be usefully leveraged to tackle individual differences on the backdrop of more general principles of brain organization. We, therefore, believe that our proposed approach of investigating shared emotion responses in relation to precisely defined dimensions of film content will lay the groundwork of understanding emotion experiences better in the broader context, including idiosyncratic determinants. This is an important step to then build upon by considering individual differences.

## Get out

Emotions serve as an interface between a person and their environment. Naturalistic films in fMRI present the opportunity to observe emotion experience more authentically and dynamically than with traditional experimental paradigms. Furthermore, we can investigate neural processes underlying emotion that are shared between individuals irrespective of their differences. We have outlined a detailed guide of a research process beginning with an explicit consideration of the theoretic constructs at play to guide annotation of film content, fMRI analysis and interpretation of findings. Specifically, our review provides an overview of the emerging imaging methodologies that might be fruitfully applied to fMRI paradigms to study emotion phenomena in humans, highlighting their possible benefits as well as their constraints and limitations. In doing so, we also call for a shift in experimental paradigms toward more task-free and naturalistic designs, such as film-based measurements, which, in turn, may require great care in stimulus selection and specification, for example by employing multidimensional annotations and investigating theory-driven constructs.

Ultimately, the goal of affective neuroscience is to integrate behavioral and neural measurements to derive a framework of brain function accounting for emotion experience. It is generally assumed that the brain gives rise to behaviors and mental states through dynamic and spatially distributed activation and connectivity. In much the same way, emotion theories predicting emotions imply the recruitment and coordination of multiple components, causally interconnected and changing over time. Yet, two major issues have slowed the integration of emotion theory and neuroscience.

Firstly, emotion theories often lack operational concepts from which testable hypotheses could be derived for neuroscience. For example, the dynamic nature of emotion is undisputed across theoretic accounts; yet, this aspect is difficult to test empirically. Most theories lack explicit predictions regarding temporality, including time scale, chronological sequence and the causal direction of relationships between components. In much the same way, often the relationships between components or emotions are described as complex and multidimensional, but the precise organization of these relationships remains elusive. There are, however, a number of psychometric models that can be used to operationalize emotion and we advocate for a closer relationship between these and emotion theory. In addition, there is a growing attempt to produce frameworks that integrate different emotion theories (Lange *et al.*, 2020).

Secondly, affective neuroscience often pays little attention to mechanistic details and information processing principles elaborated by emotion theories (Lewis, 2005). Though psychological models underscore the dynamic and complex interaction of various factors that give rise to emotion, many neuroscience studies still content with very simple analyses, sometimes limited to merely localizing brain activation related to different emotions and/or aspects of emotions, conceptualized in a relatively monolithic and static manner. Research with greater computational efforts, in turn, often ignores the manifold constituents of emotion or considers few variables at a time (Saarimäki, 2021).

We believe that time is ripe for a more systematic approach to study the neural underpinnings of emotion using film fMRI and leverage innovative imaging analysis techniques that can be deployed in this context, while conversely the advent of these novel methodologies may allow a fresh look and exciting new approaches to the study of emotion. To this aim, a stronger dialogue needs to take place between emotion theorists and affective neuroscientists, so that theory and neuroscience can be more integrated (Lewis, 2005). To make empirical studies interpretable and relevant for theory development, neuroscientists need to include considerations from emotion theory at the planning and design stage of research studies, so that they can test (or falsify) specific hypotheses derived from such theories. Furthermore, to get a better understanding of how well a given theory is supported by data, it will be important to integrate assumptions from multiple theoretic accounts. This may also provide a space in which features of different theories can be consolidated, confronted or integrated to improve our mechanistic understanding of emotion processes in line with empirical findings. Such efforts to combine elements of different theories can provide a basis for further empirical studies testing alternative frameworks and interpreting conflicting findings more easily. Ultimately, appropriate analysis techniques must be employed that are anchored in specific research questions and can produce clear and interpretable results.

Throughout our review, we have given some general guidance as to the theoretic implications of different analytical approaches. However, these do not constitute a prescription of precise algorithms or ‘recipes’, and we recommend to decide on a case-by-case basis which methods are a good fit for a certain research question. Many times, this will mean combining multiple analysis techniques to probe different aspects of emotion. In addition, one might consider multimodal imaging techniques to combine the spatial resolution of fMRI with the temporal resolution of other techniques.

There is much potential in film fMRI to further our understanding of brain function. While this review is specifically aimed at emotion research, we believe that researchers seeking to study other phenomena with film fMRI will also benefit from it. Nevertheless, film fMRI is not a replacement for task fMRI, but a complementary method in a cycle of scientific experimentation (Matusz *et al.*, 2019). Findings from film fMRI have the potential to generate new questions that may, in turn, be answered using more traditional designs, and vice versa. Current theories might be misled by various a priori conceptions of what emotions are, which may or may not accord with how they are actually implemented in neural terms. For this reason, we believe film fMRI (and other approaches allowing interactive first-person implication such as gaming or VR) might constitute a powerful discovery tool to describe emotion phenomena in new ways with a bottom-up, data-driven brain perspective, eventually leading to reformulate emotion concepts and theories. On the other hand, there are research questions that lend themselves to more or less naturalistic research paradigms. So, while we need task fMRI, film and other naturalistic paradigms offer an integral and powerful tool in the future of neuroscience (Finn, 2021). This will prompt the development of innovative analysis techniques, allowing us to better study how the brain works in real life rather than just in lab paradigms.

There is much room for progress in this field and we very much hope that this will be community driven. As with most of neuroscience, this is very resource-intensive work. Therefore, continued willingness to collaborate and share data, code and expertise will make a substantial difference to the future of this research.

## Data Availability

No new data were generated or analysed in support of this review paper.
